# Single-cell analysis of microglial activation after traumatic brain injury reveals immune signaling pathways linked to mitochondrial dysfunction and brain aging

**DOI:** 10.3389/fnagi.2025.1657523

**Published:** 2026-01-09

**Authors:** Ming Sun, Chao Wu, Jingjing Wu, Lixin Liu, Liang Gu, Zihao Wang, Xue Yang, Feng Xu

**Affiliations:** 1 Department of Emergency Medicine, The Affiliated Suqian Hospital of Xuzhou Medical University, Suqian, China; 2 Suqian Medical Research Institute of Nanjing University Medical School, Suqian, China; 3 Department of Emergency Medicine, Suqian Clinical Medical College of Yangzhou University, Suqian, China; 4 Department of Emergency Medicine, Nanjing Drum Tower Hospital Group Suqian Hospital, Suqian, China; 5 Department of Hyperbaric Oxygen Medicine, Shenzhen Children’s Hospital, Shenzhen, China; 6 Department of Emergency Medicine, the First Affiliated Hospital of Soochow University, Suzhou, China

**Keywords:** microglial activation, myeloid response, neuroinflammation, single-cell transcriptomics, traumatic brain injury

## Abstract

**Objective:**

Microglia are the primary immune cells in the central nervous system (CNS); however, their temporal and spatial responses to traumatic brain injury (TBI) at the single-cell level remain poorly defined. This study aimed to map the dynamic microglial responses to TBI using single-cell transcriptomics and validate key signaling pathways *in vitro*.

**Methods:**

A single-cell transcriptomic atlas was reconstructed from publicly available datasets comprising cortical, hippocampal, and blood samples from 35 mice (11 blood, 12 cortex, and 12 hippocampus) subjected to TBI or sham treatment at 24 h and after 7 days. Comparative analyses were conducted to investigate the heterogeneity of myeloid cells, including monocytes, macrophages, and microglia, with a particular focus on activated microglia. The key findings were further validated using quantitative PCR (qPCR) in an *in vitro* TBI-mimicking model, employing lipopolysaccharide (LPS)-stimulated microglial cell lines to assess changes in gene expression.

**Results:**

TBI induced rapid immune remodeling, including an increase in activated microglia in the cortex, enriched in leukocyte differentiation pathways, and elevated macrophage populations in the cortex and hippocampus, enriched in chemotaxis functions at 24 h. Ligand–receptor (LR) analysis revealed three major signaling axes—Ccl2/Ccl7–Ccr2, Tnf–Tnfrsf1b, and Grn–Flna—associated with monocyte recruitment, M1 polarization, and macrophage differentiation. Validation using qPCR confirmed significant upregulation of Ccl2, Tnf, and Grn in LPS-stimulated microglia, which is consistent with single-cell findings.

**Conclusion:**

This study provides the first integrative single-cell transcriptomic map of microglial–myeloid interactions after TBI across multiple tissues and time points, linking microglial signaling to mitochondrial dysfunction and neuroinflammation. These findings lay the foundation for therapeutic strategies targeting myeloid-driven immune regulation in TBI.

## Introduction

Traumatic brain injury (TBI) is a complex neurological disorder caused by external mechanical forces, resulting in both immediate neuronal damage and long-term functional impairments. In addition to acute neuronal injury, TBI triggers a multifaceted neuroinflammatory response in the central nervous system (CNS), which significantly influences disease progression, tissue repair, and recovery. The long-term consequences of TBI include cognitive deficits, memory impairment, motor dysfunction, and behavioral disturbances, contributing to substantial morbidity and reduced quality of life (Blennow et al., 2016). TBI is a global public health concern, affecting millions annually, with many patients developing chronic neurodegenerative disorders such as Alzheimer’s disease or chronic traumatic encephalopathy (Jessen, 2004). Central to TBI-induced neuroinflammation are glial cells, particularly microglia, the resident immune population of the CNS and the first responders to injury. Microglia continuously monitor the brain’s environment and respond rapidly to pathogens, injury, or stress by altering their morphology, gene expression, and functional properties (Colonna and Butovsky, 2017). Upon activation, microglia release pro-inflammatory mediators, such as cytokines (IL-1β, TNF-α), chemokines, and reactive oxygen species (ROS), which recruit peripheral immune cells, clear debris, and maintain blood–brain barrier integrity (Kettenmann et al., 2013; Lampron et al., 2013). Although this response is protective in the short term, prolonged or excessive activation exacerbates inflammation, damages neurons, and contributes to secondary injury (Xu et al., 2017; Madathil et al., 2018). A key feature of microglial activation is their polarization into distinct functional phenotypes. The pro-inflammatory M1 phenotype promotes the release of cytokines such as IL-1β, TNF-α, and IFN-γ, which can drive neurotoxicity, whereas the anti-inflammatory M2 phenotype supports tissue repair and the resolution of inflammation through IL-10 and TGF-β signaling (Ott et al., 1994; Kim et al., 2015; Yang and Zhou, 2019). The balance between these phenotypes critically influences the outcomes of TBI, The predominance of the M1 phenotype is associated with sustained injury and poor recovery, while M2 activity promotes repair. Furthermore, interactions between microglia and infiltrating myeloid cells, including monocytes and macrophages, shape the overall immune response. Peripheral monocytes infiltrate the injured brain, differentiate into macrophages, and contribute to both inflammatory and reparative processes, mediated by signaling pathways such as CCL2/CCR2, CCL7/CCR2, and TNF/TNFRSF1B (D’Mello et al., 2009; Shechter et al., 2011; Gschwandtner et al., 2019). Mitochondrial dysfunction is increasingly recognized as a central modulator of microglial activation, particularly in aging. Impaired mitochondrial dynamics, altered energy metabolism, and increased oxidative stress bias microglia toward a pro-inflammatory M1 state, exacerbating neuroinflammation and hindering recovery following TBI. This mechanistic link provides a potential explanation for age-related vulnerability to TBI and neurodegenerative progression. Despite extensive research, much of our current understanding relies on bulk tissue analyses, which obscure cellular heterogeneity and dynamic immune responses. Single-cell RNA sequencing (scRNA-seq) provides high-resolution insights into the diversity of microglial and myeloid cells, revealing temporal and spatial activation patterns following injury.

In this study, we hypothesize that TBI induces dynamic, tissue-specific microglial activation states that interact with infiltrating myeloid cell populations through defined signaling axes. We further propose that these responses are modulated by mitochondrial function and aging. To test this hypothesis, we reconstructed a multi-tissue single-cell RNA sequencing (scRNA-seq) atlas of TBI in mice and validated key pathways *in vitro*, aiming to elucidate cellular and molecular mechanisms underlying post-TBI neuroinflammation and identify potential therapeutic targets.

## Materials and methods

This study was conducted according to the laboratory standards of the First Affiliated Hospital of Suqian, Xuzhou Medical University. Ethical approval was not required because the study utilized only publicly available transcriptomic data and commercially available standard cell lines. No human participants or animals were directly involved.

### Data retrieval and single-cell atlas reconstruction

Single-cell RNA sequencing (scRNA-seq) barcode, features, matrix.mtx, and metadata files were downloaded from the Gene Expression Omnibus (GEO) database^1^ under accession number GSE180862. This dataset comprises mouse cortical, hippocampal, and peripheral blood samples from a controlled cortical impact (CCI)-based TBI model at 24 h and 7 days post-injury, generated using the 10x Genomics Chromium platform with Illumina sequencing. A total of 35 mice were included (11 blood, 12 cortex, and 12 hippocampus). Raw barcode, features, and matrix files were downloaded and processed in R (v4.2.1) using the Seurat package (Hao et al., 2021). Quality control included the exclusion of cells with <200 or >6,000 detected genes or with >10% mitochondrial gene expression. A “LogNormalize” scaling method was used for each Seurat object, followed by a “FindVariableFeatures” step using the “vst” method and “nfeatures” of 2,000. Principal component analysis (PCA) was used for linear dimensional reduction, and the Uniform Manifold Approximation and Projection (UMAP) method was used for the visualization of cellular plots. Subgroup information was retrieved from “metadata” files. To minimize technical artifacts arising from different tissue types (blood, cortex, hippocampus), independent Seurat objects were created for each tissue instead of integrating across compartments. This approach preserves biologically meaningful inter-tissue heterogeneity in immune composition, consistent with prior single-cell injury atlases in which integration may obscure compartment-specific signatures (Schafflick et al., 2020; Tran et al., 2020).

### Trajectory inference using pseudotime analysis

Cellular differentiation dynamics were inferred using Monocle with the R “monocle” package (Trapnell et al., 2014). Overlapping features across tissues were extracted, and trajectory trees were reconstructed for blood Ly6c^+^ monocytes, Treml4^+^ monocytes, and CNS-resident MG/aMG cells. Genes with branch-dependent expression were visualized using branched heatmaps. Different trajectory trees were visualized using the “plot_cell_trajectory” function. Branch-featured genes were summarized using the R “pheatmap” package and visualized using the “plot_genes_branched_heatmap” function.

### Gene Ontology biological process enrichment analysis

Gene Ontology (GO) enrichment analysis was performed using the R “clusterprofiler” package (Wu et al., 2021). The “enrichGO” function was used to calculate enriched GO biological process terms, and a *p*-adjust value of 0.05 was used as a significance cutoff.

### Ligand–receptor interaction screening

Protein–protein interaction information was downloaded from the BioGRID database (Stark et al., 2006) under the Mus.mucus (101574) term. Subcellular location information for the involved proteins was retrieved from the UniProt website.^2^ A ligand–receptor (LR) pair was screened only if it satisfied all of the following criteria: (1) gene A belonged to subgroup 1, 2, 3 or 6; (2) gene A was annotated as “secreted;” (3) gene B belonged to subgroup 4 or 5; (4) gene B was annotated as “plasma membrane;” and (5) A and B interacted with each other.

### Gene set variation analysis

Gene set variation analysis (GSVA) was performed using the R package “GSVA” (Hänzelmann et al., 2013). Cells defined as Ly6c^+^.Mon andTreml4^+^.Mon from the blood samples, as well as MAC, MG, and aMG cells from the cortex and hippocampus samples, were involved in GSVA analysis. Log2-scaled expression data were used as input. Student’s *t*-test was used to compare the activation states of specific pathways between different groups, and a *p*-value of 0.05 was used as a significance cutoff.

### Validation *in vitro*

To validate the transcriptomic findings, we employed an *in vitro* neuroinflammation model using the murine BV2 microglial cell line (Procell Life Science & Technology Co., Ltd., Wuhan, China). BV2 cells were cultured in Dulbecco’s Modified Eagle Medium (DMEM; Gibco) supplemented with 10% fetal bovine serum (FBS; Gibco) and 1% penicillin–streptomycin (Gibco) at 37 °C in a humidified atmosphere containing 5% CO₂. The cells were seeded in 6-well plates at a density of 1 × 10^6^ cells/well and allowed to adhere overnight. To simulate TBI-associated neuroinflammation, the BV2 cells were treated with lipopolysaccharide (LPS; Sigma-Aldrich) at a concentration of 100 ng/mL for 24 h. This concentration and exposure duration were selected based on prior studies demonstrating robust induction of pro-inflammatory cytokines and microglial activation without excessive cytotoxicity (Hongxia et al., 2019; An et al., 2020). Untreated cells served as negative controls. Total RNA was extracted using TRIzol reagent (Invitrogen) following the manufacturer’s instructions. RNA concentration and purity were assessed using a NanoDrop spectrophotometer. Complementary DNA (cDNA) was synthesized using the PrimeScript RT Reagent Kit (Takara) according to the manufacturer’s instructions. qPCR was performed using SYBR Green Master Mix (Applied Biosystems) on a QuantStudio 5 platform (Thermo Fisher Scientific). Target genes (Ccl2, Tnf, and Grn) were normalized to GAPDH expression. Relative expression was calculated using the 2^−ΔΔCT^ method. All qPCR experiments included three independent biological replicates; each measured in technical triplicates. Data were expressed as mean ± standard deviation (SD). Group differences were assessed using an unpaired two-tailed Student’s *t*-test, with the Benjamini–Hochberg correction for multiple comparisons applied where appropriate.

## Results

### Reconstruction of mouse tissue-specific single-cell atlas

**Figure 1:**
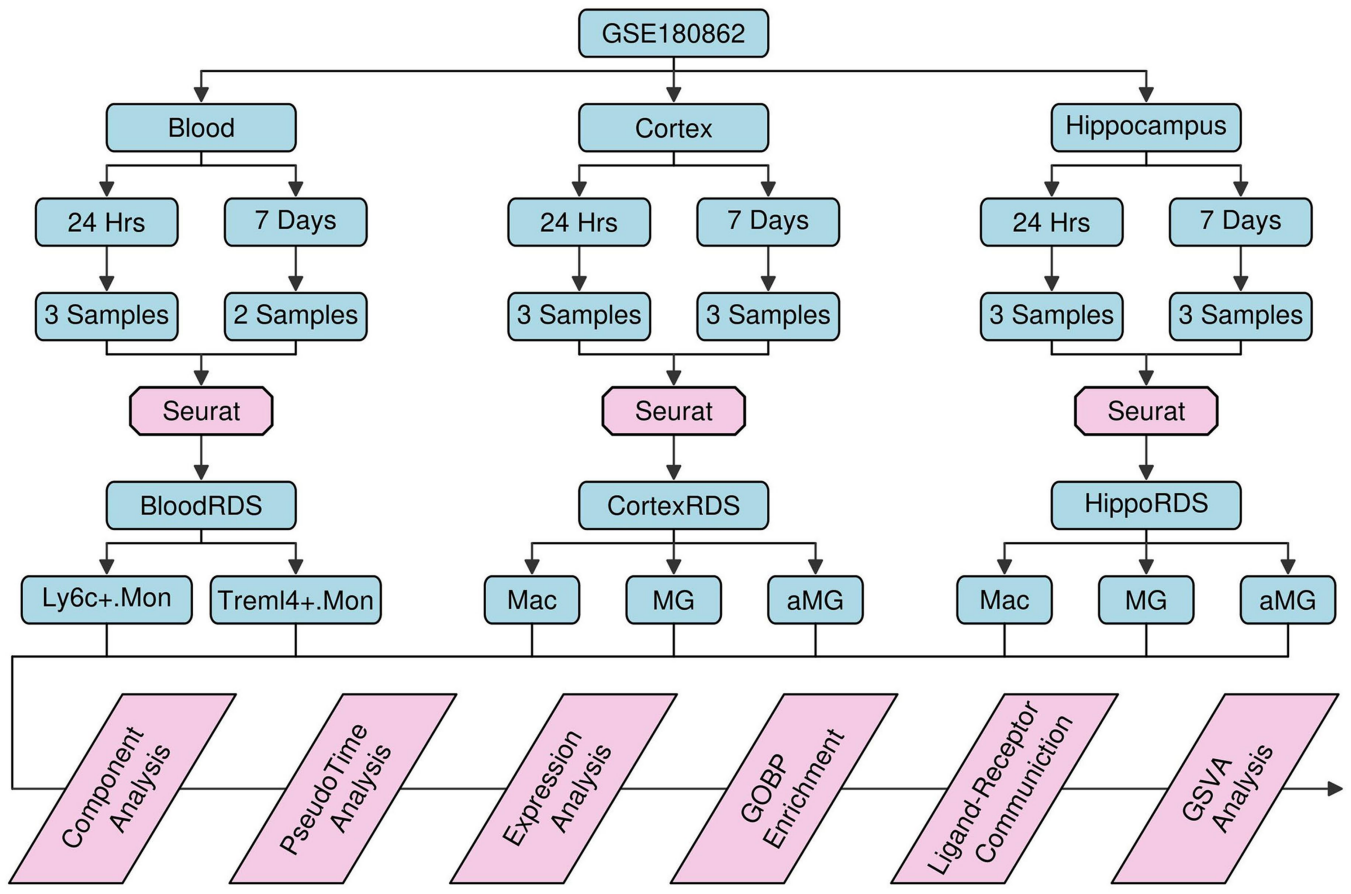
Flowchart illustrating the study design.

**Figure 2:**
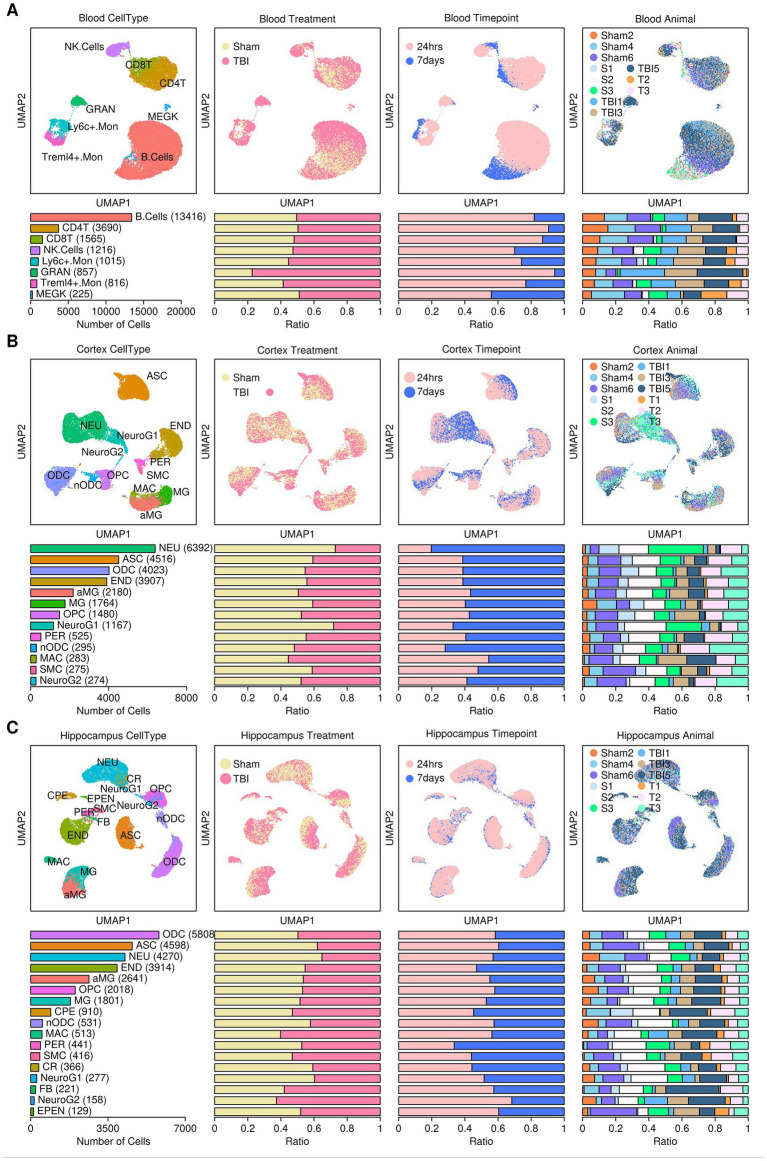
Reconstruction of the mouse single-cell atlas. **(A)** Blood samples. **(B)** Cortex samples **(C)** Hippocampus samples. Top panel from left to right: UMPA plot showing the distribution patterns of subgroups, treatment conditions, sampling time points, and mouse IDs. Bottom panel from left to right: Bar plot representing the number of cells in each subgroup, the relative proportion of cells under different treatment conditions, the relative proportion of cells at different sampling time points, and the relative proportion of cells from each mouse. B. cells, B cells; CD4T, CD4^+^ T cells; CD8T, CD8^+^ T cells; NK cells, natural killer cells; Ly6c^+^.Mon, Ly6c^+^ monocytes; GRAN, granulocytes; Treml4^+^.Mon, Treml4^+^ monocytes; MEGK, megakaryocytes; NEU, neurons; ASC, astrocytes; ODC, oligodendrocytes; END, endothelial cells; aMG, activated microglia; MG, microglia; OPC, oligodendrocyte precursors; NeuroG1, neurogenesis1 cells; PER, pericytes; nODC, new oligodendrocytes; MAC, macrophages; SMC, smooth muscle cells; NeuroG2, neurogenesis2 cells; CPE, choroid plexus epithelial cells; CR, Cajal–Retzius cells; FB, fibroblasts; EPEN, ependymal cells.

We first reconstructed a single-cell atlas of mouse tissues subjected to TBI or sham control treatments using single-cell matrix files retrieved from the GEO database (GSE180862) (Arneson et al., 2022). A brief flowchart illustrating the research design of this study is shown in Figure 1. To eliminate the impact of batch effects, we performed scRNAseq analysis for samples from different tissue sources rather than integrating them. Regarding cells from the blood samples, a single-cell atlas containing 22,800 cells was constructed from 11 mice (Figure 2A), and these cells were further clustered into eight subgroups based on the relative expression levels of their marker genes: B. cells (B cells, 13,416 cells, 58.84%), CD4T (Cd4^+^ T cells, 3,690 cells, 16.18%), CD8T (Cd8^+^ T cells, 6.86%), NK. Cells (Natural killer cells, 1,216 cells, 5.33%), Ly6c^+^.Mon (Ly6c^+^ monocytes, 1,015 cells, 4.45%), GRAN (Granulocytes, 857 cells, 3.76%), Treml4^+^.Mon (Treml4^+^ monocytes, 816 cells, 3.58%), and MEGK (Megakaryocytes, 225 cells, 0.99%). Plots representing the sample origins (including treatment types: TBI vs. sham control; timepoints: 24 h vs. 7 days; 11 mice) across the different subgroups are also shown in Figure 2A. From the cortex samples, a single-cell atlas containing 27,081 cells was constructed from 12 mice (Figure 2B). These cells were further clustered into 13 subgroups: NEU (neurons, 6,392 cells, 23.60%), ASC (astrocytes, 4,516 cells, 16.68%), ODC (oligodendrocytes, 4,023 cells, 14.85%), END (endothelial cells, 3,907 cells, 14.43%), aMG (activated microglia cells, 8.05%), MG (microglia cells, 1,764 cells, 6.51%), OPC (oligodendrocyte precursors, 1,480 cells, 5.47%), NeuroG1 (neurogenesis1 cells, 1,167 cells, 4.31%), PER (pericytes, 525 cells, 1.94%), nODC (new oligodendrocytes, 295 cells, 1.09%), MAC (macrophages, 283 cells, 1.05%), SMC (smooth muscle cells, 275 cells, 1.02%), and NeuroG2 (neurogenesis2 cells, 274 cells, 1.01%). Plots representing the sample origins (including treatment types: TBI vs. sham control; timepoints: 24 h vs. 7 days; 12 mice) across the different subgroups are also shown in Figure 2B. From the hippocampus samples, a single-cell atlas containing 29,012 cells was constructed from 12 mice (Figure 2C). These cells were further clustered into 17 subgroups: ODC (oligodendrocytes, 5,808 cells, 20.19%), ASC (astrocytes, 4,598 cells, 15.85%), NEU (neurons, 4,270 cells, 14.37%), END (endothelial cells, 3,914 cells, 13.49%), aMG (activated microglia cells, 2,641 cells, 9.10%), OPC (oligodendrocyte precursors, 2,018 cells, 6.96%), MG (microglia cells, 1,801 cells, 6.21%), CPE (choroid plexus epithelial cells, 910 cells, 3.14%), nODC (new oligodendrocytes, 531 cells, 1.83%), MAC (macrophages, 513 cells, 1.77%), PER (pericytes, 441 cells, 1.52%), SMC (smooth muscle cells, 416 cells, 1.43%), CR (Cajal–Retzius cells, 366 cells, 1.26%), NeuroG1 (neurogenesis1 cells, 277 cells, 0.95%), FB (fibroblasts, 221 cells, 0.76%), NeuroG2 (nerogenesis2 cells, 158 cells, 0.54%), and EPEN (ependymal cells, 129 cells, 0.44%). Plots representing the sample origins (including treatment types: TBI vs. sham control; timepoints: 24 h vs. 7 days; 12 mice) across the different subgroups are also shown in Figure 2C.

### Dynamics of myeloid-related components in different tissues after TBI treatment

**Figure 3:**
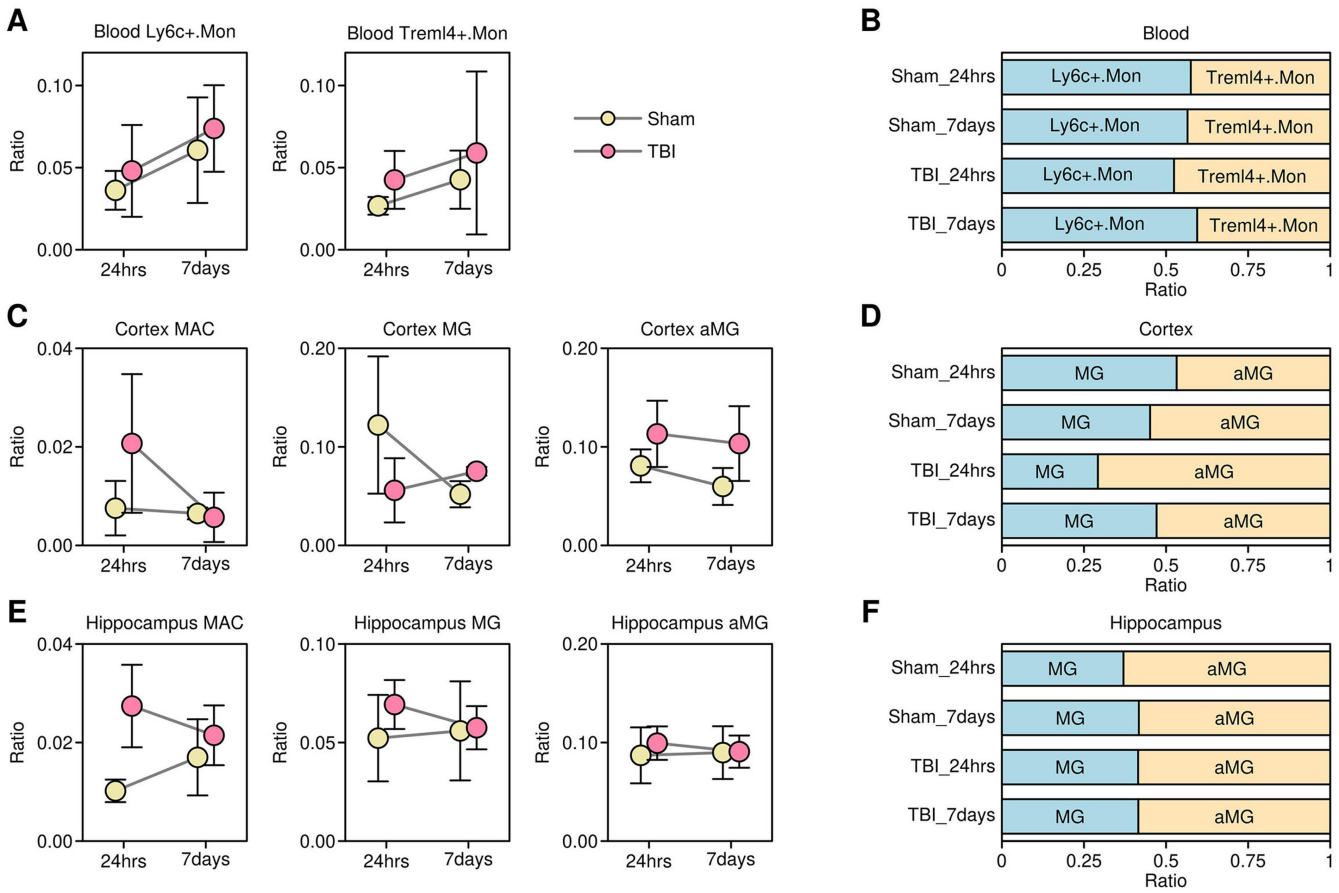
Dynamics of myeloid-related components. **(A)** Dot plot showing changes in the proportions of monocyte subgroups in blood samples. **(B)** Bar plot showing changes in the proportions of Ly6c^+^.Mon and Treml4^+^.Mon across different samples. **(C)** Dot plot showing changes in the proportions of macrophage and microglia subgroups in cortex samples. **(D)** Bar plot showing changes in the proportions of microglia versus activated microglia across different samples. **(E)** Dot plot showing changes in the proportions of macrophage and microglia subgroups in hippocampus samples. **(F)** Bar plot showing changes in the proportions of microglia versus activated microglia across different samples.

Innate immunity plays an important role in the clearance of cellular debris and tissue repair (Fani Maleki and Rivest, 2019). To investigate changes in innate immune components after TBI, we compared the relative proportions of monocytes (including Ly6c^+^.Mon and Treml4^+^.Mon), macrophages (MAC), microglia (MG), and activated microglia (aMG) across different sample origins. The results are presented in Figure 3. In blood tissues, there was a slight increase in the proportions of both Ly6c^+^.Mon and Treml4^+^.Mon cells in the TBI-treated samples at 24 h and 7 days compared to the sham-treated samples (Figure 3A). Interestingly, among all monocytes, a higher proportion of Treml4^+^.Mon cells was found in the TBI-treated samples at 24 h compared to the TBI-treated samples at 7 days or sham-treated samples, indicating that Treml4^+^.Mon cells may play an important role in the acute phase of TBI (Figure 3B). In the cortex tissues, there was an increase in the proportion of MAC cells in the TBI-treated samples at 24 h compared to the sham-treated samples, but this increase was no longer observed at 7 days. There was a decrease in the proportion of MG cells in the TBI-treated samples at 24 h compared to the sham-treated samples, and this decrease was nearly resolved by 7 days. There was an increase in the proportion of aMG cells in the TBI-treated samples at both 24 h and 7 days compared to the sham-treated samples (Figure 3C). Among all microglia, a higher proportion of aMG cells was found in the TBI-treated samples at 24 h, suggesting a rapid microglial activation response after TBI (Figure 3D). In hippocampus tissues, there was an increase in the proportion of MAC cells in the TBI-treated samples at 24 h compared to the sham-treated samples, but this increase was no longer observed at 7 days. There were almost no changes in the proportions of MG and aMG cells between the TBI-treated samples and sham-treated control samples at 24 h or 7 days (Figure 3E). Among all microglia, there was a slight increase in the proportion of aMG cells in the sham-treated control samples at 24 h (Figure 3F).

### Evolutionary relationship among different myeloid components revealed via pseudotime analysis

**Figure 4:**
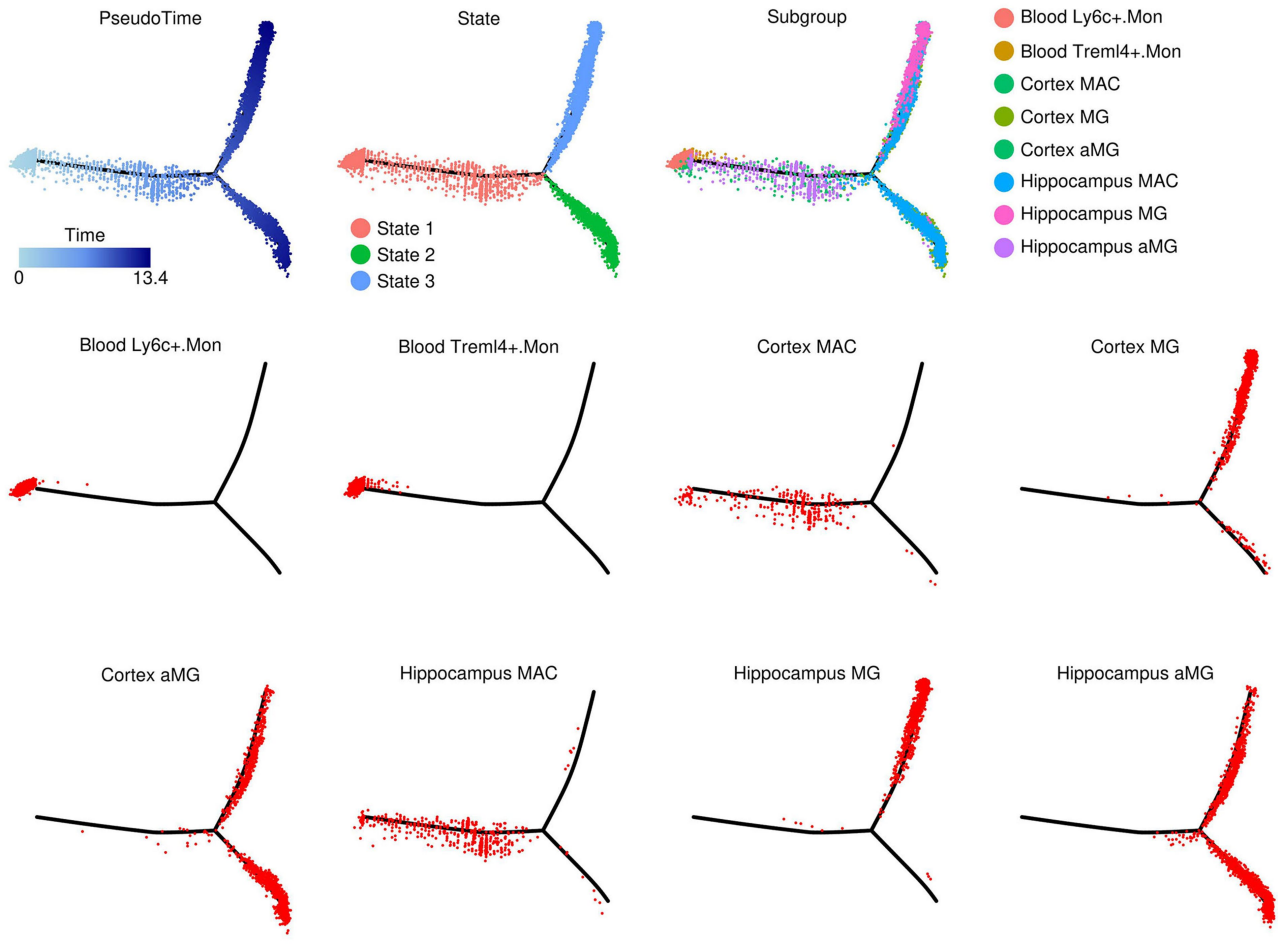
Evolutionary relationships of myeloid components revealed through pseudotime analysis.

**Figure 5:**
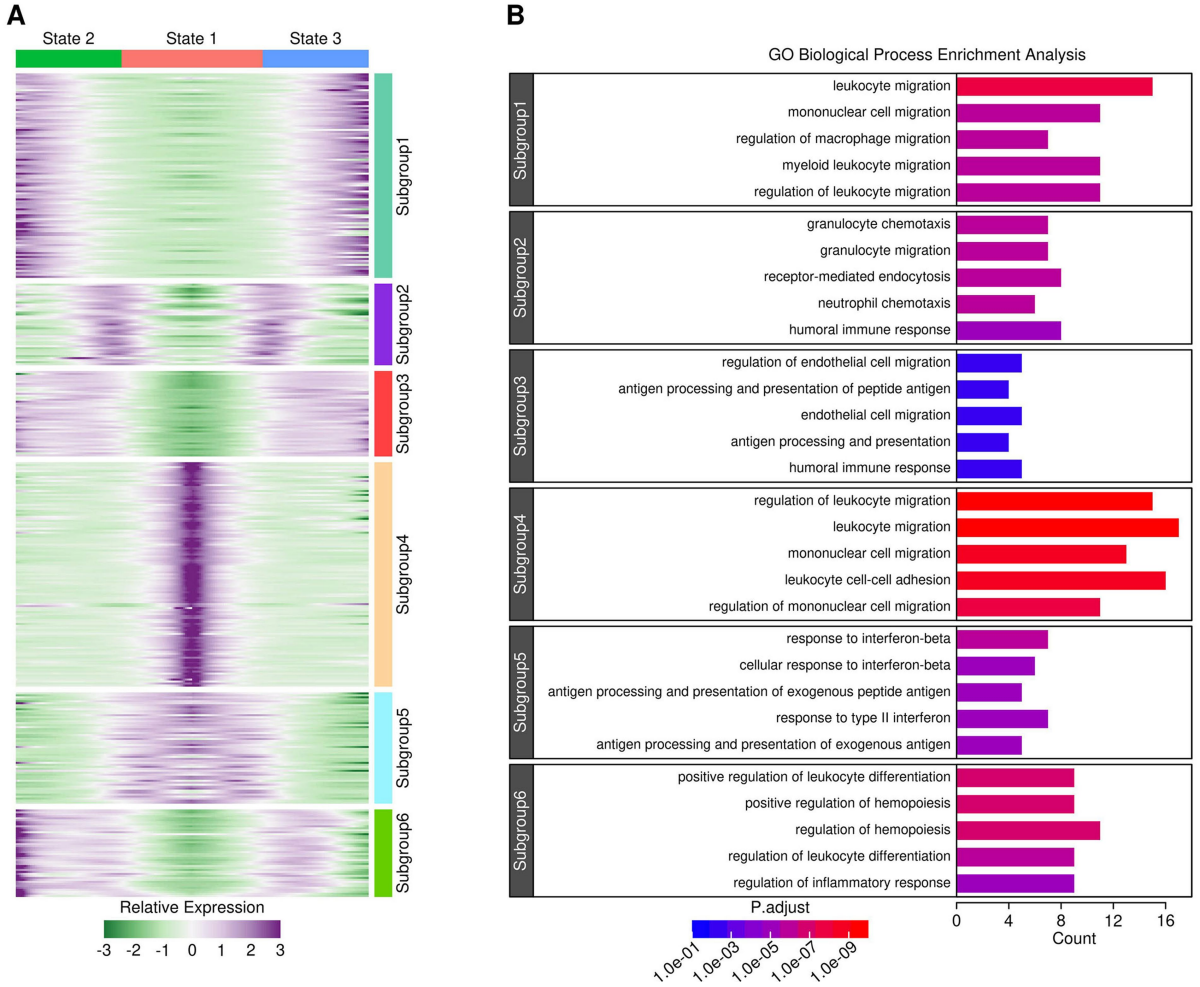
Gene expression profiles across cells from different branches. **(A)** Heatmap showing gene expression patterns across cells from different branches. **(B)** GO biological process enrichment results for genes from each subgroup. A *p*-adjust value of 0.05 was used as a significance cutoff.

The evolutionary relationship among different myeloid components was investigated using the R “monocle” package, and the results are presented in Figure 4. After pseudotime analysis, a clear three-state trajectory was identified (Tree “PsudoTime” and “State”). Most of the monocytes and macrophages resided in the state 1 branch, with monocytes inferred to be the root of the three-branch trajectory (Tree “Blood Ly6c^+^.Mon,” “Blood Treml4^+^.Mon,” “Cortex MAC,” and “Hippocampus MAC”). Most microglia resided in the state 3 branch (Tree “Cortex MG” and “Hippocampus MG”), while most activated microglia resided in the state 2 branch (Tree “Cortex aMG” and “Hippocampus aMG”), suggesting an activation trajectory of these microglia. A further comparison of gene expression profiles across cells from these three branches identified six gene subgroups with different expression patterns, as shown in Figure 5A, and the top-enriched GO biological processes of genes from these six subgroups are listed in Figure 5B. Genes from subgroups 1, 2, and 3 were highly expressed in state 2 and state 3 cells (microglia and activated microglia). Specifically, subgroup 1 genes were enriched in “regulation of macrophage migration,” subgroup 2 genes were enriched in “neutrophil and granulocyte chemotaxis,” and subgroup 3 genes were enriched in “regulation of epithelial cell migration.” Genes from subgroup 4 were highly expressed in monocytes (Ly6c^+^.Mon and Treml4^+^.Mon cells), and these genes were enriched in “leukocyte cell–cell adhesion” and “mononuclear cell migration.” Genes from subgroup 5 were highly expressed in macrophages (Cortex MAC and Hippocampus MAC cells), and these genes were enriched in “response to inferno-gamma.” Genes from subgroup 6 were highly expressed in activated microglia (Cortex aMG and Hippocampus aMG cells), and these genes were enriched in “regulation of leukocyte differentiation” and “regulation of inflammatory response.”

### Ligand–receptor communications between microglia cells and monocytes/macrophages

**Figure 6:**
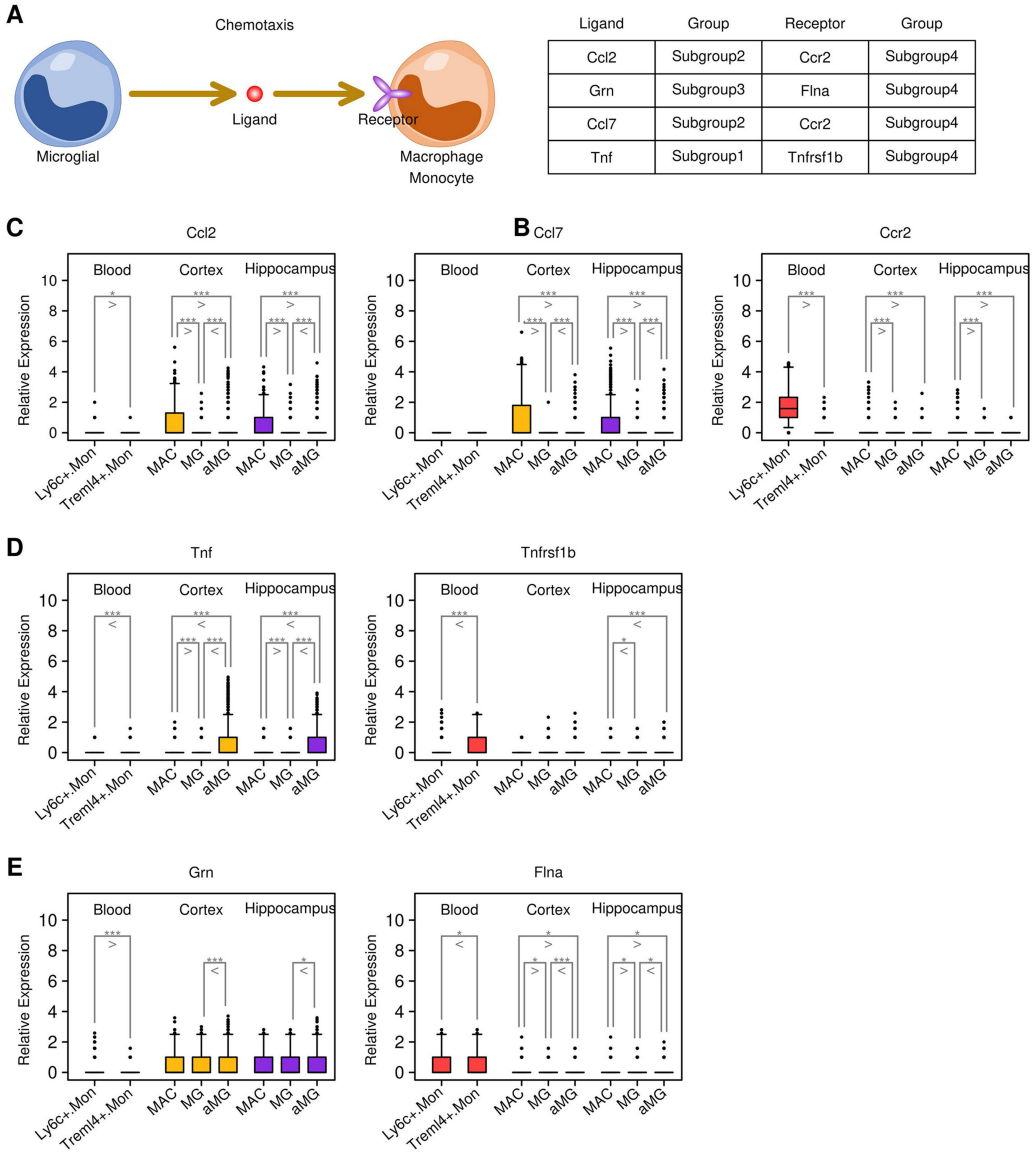
Key communications identified through ligand–receptor screening. **(A)** Cartoon map illustrating the ligand–receptor screening process. **(B)** List of key communications between genes from different subgroups. **(C)** Expression status of the *Ccl2/Ccl7/Ccr2* pair across different subgroups. **(D)** Expression status of the *Tnf/Tnfrsf1b* pair across different subgroups. **(E)** Expression status of the *Grn/Flna* pair across different subgroups. A Student’s *t*-test was performed for each comparison (^*^p < 0.05, ^**^p < 0.01, and ^***^p < 0.001).

**Figure 7:**
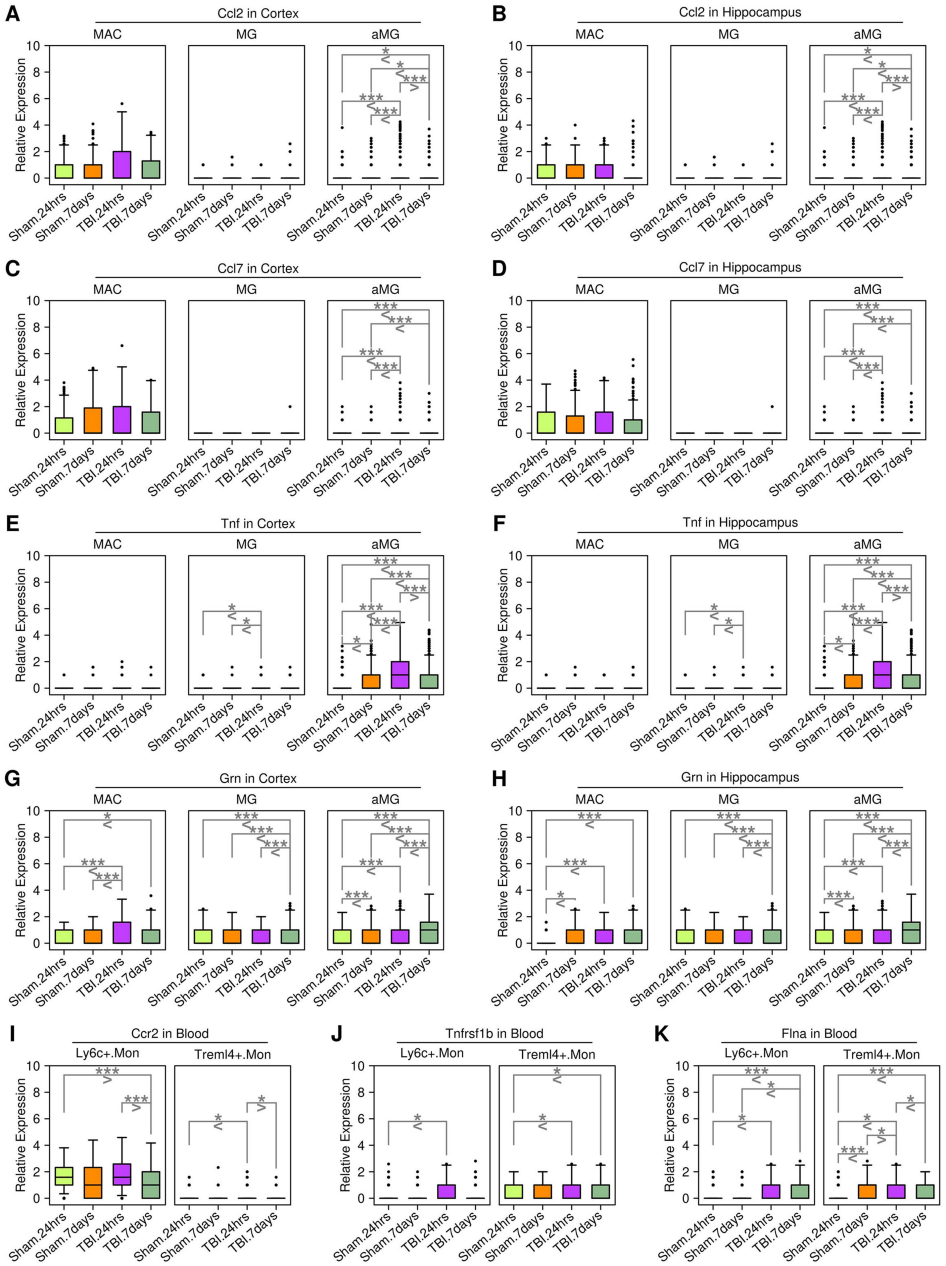
Expression patterns of key genes in cells from different sources. **(A)** Expression levels of the *Ccl2* gene in cortex samples. **(B)** Expression levels of the *Ccl2* gene in hippocampus samples. **(C)** Expression levels of the *Ccl7* gene in cortex samples. **(D)** Expression levels of the *Ccl7* gene in hippocampus samples. **(E)** Expression levels of the *Tnf* gene in cortex samples. **(F)** Expression levels of the *Tnf* gene in hippocampus samples. **(G)** Expression levels of the *Grn* gene in cortex samples. **(H)** Expression levels of the *Grn* gene in hippocampus samples. **(I)** Expression levels of the *Ccr2* gene in blood samples. **(J)** Expression levels of the *Tnfrsf1b* gene in blood samples. **(K)** Expression levels of the *Flna* gene in blood samples. A Student’s *t*-test was performed for each comparison (^*^p < 0.05, ^**^p < 0.01, and ^***^p < 0.001).

Secreted ligands represent one of the most direct mechanisms by which target cells communicate with other cells. In this study, we further investigated ligand–receptor (LR) interactions between microglial cells and macrophages/monocytes (illustrated in Figure 6A), and the results are presented in Figure 6B. Together, we identified four LR interactions: *Ccl2*-*Ccr2*, *Ccl7-Ccr2*, *Tnf-Tnfrsf1b*, and *Grn-Flna*. Specifically, *Ccl2* and *Ccl7* genes were highly expressed in cortex and hippocampus MG cells, and the expression levels of *Ccl2* and *Ccl7* were higher in aMG cells compared to MG cells. The *Ccr2* gene was highly expressed in Ly6c^+^.Mon cells from the blood samples (Figure 6C). The *Tnf* gene was highly expressed in cortex and hippocampus aMG cells, and the *Tnfrsf1b* gene was highly expressed in Treml4^+^.Mon cells (Figure 6D). The expression of *Grn* was also upregulated in aMG cells compared to MG cells, and the *Flna* gene was highly expressed in monocytes (Figure 6E). We further examined the expression of these genes in subgroups from different sample sources, and the results are presented in Figure 7. After 24 h of TBI treatment, *Ccl2* expression was significantly increased in aMG cells from the cortex samples (Figure 7A) and MG and aMG cells from the hippocampus samples (Figure 7B). Similarly, *Ccl7* expression was significantly increased in aMG cells from the cortex samples (Figure 7C) and MG and aMG cells from the hippocampus samples (Figure 7D). There was a significant increase in the expression of *Ccr2* in Ly6c^+^.Mon cells from the blood samples (Figure 7I), suggesting a *Ccl2/Ccl7/Ccr2*-mediated monocyte chemotaxis process shortly after TBI treatment. Regarding the *Tnf–Tnfrsf1b* pair, after 24 h of TBI treatment, *Tnf* expression was significantly increased in aMG cells from the cortex samples (Figure 7E) and in MG and aMG cells from the hippocampus samples (Figure 7F). Similarly, *Tnfrsf1b* expression was significantly increased in Ly6c^+^.Mon and Treml4^+^.Mon cells from the blood samples (Figure 7J), suggesting a Tnf/Tnfrsf1b-mediated M1 macrophage polarization process shortly after TBI treatment. Regarding the *Grn–Flna* pair, 7 days after TBI treatment, *Grn* expression was significantly increased in MG and aMG cells from both the cortex and hippocampus samples (Figures 7G,H). Similarly, *Flna* expression was significantly increased in Ly6c^+^.Mon and Treml4^+^.Mon cells from the blood samples (Figure 7K), suggesting a delayed regulatory effect mediated through the *Grn–Flna* pair after TBI treatment.

### Upregulated biological processes in activated microglia

**Figure 8:**
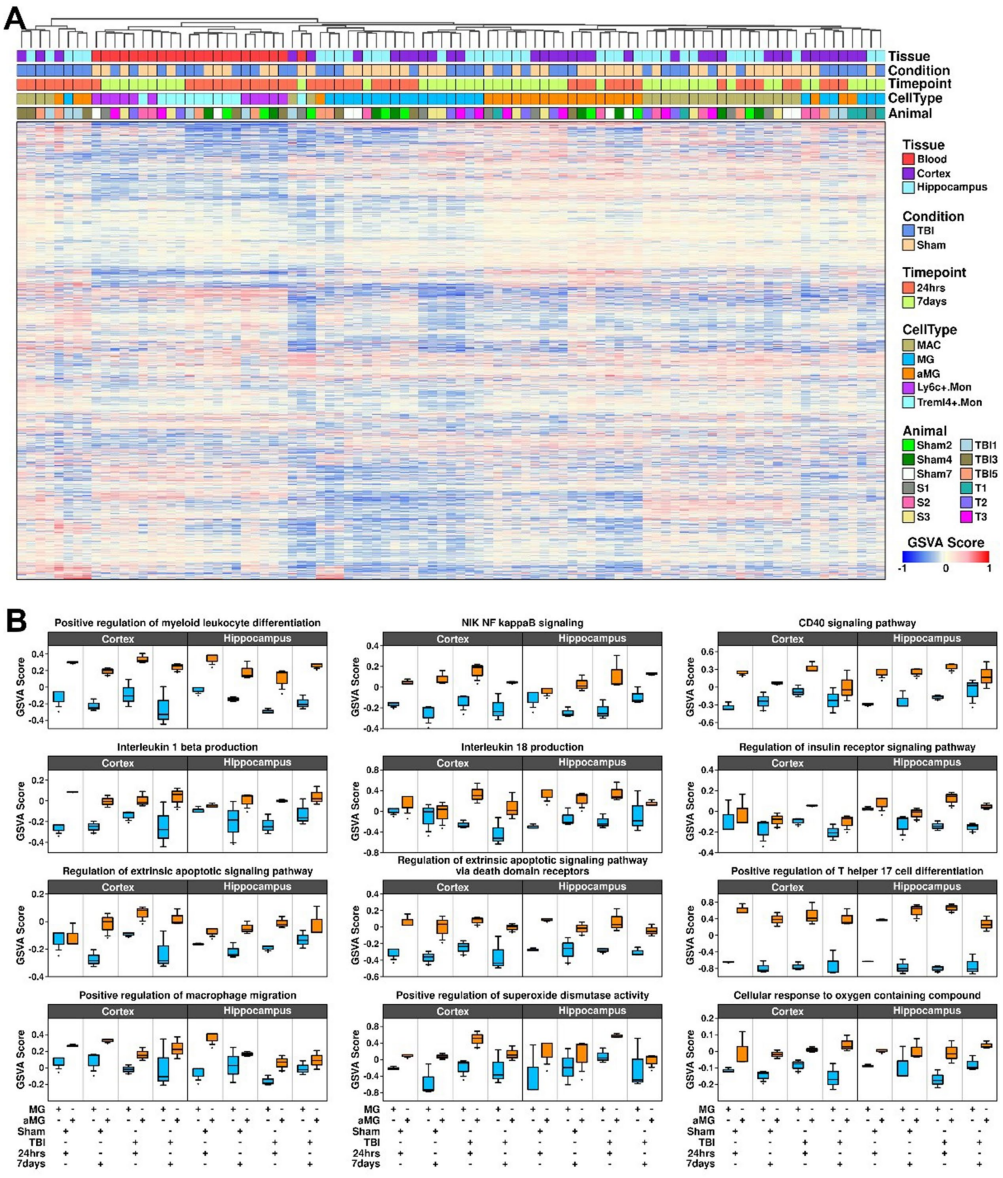
Upregulated biological pathways identified in activated microglia. **(A)** Heatmap showing GSVA results of GO biological pathways in myeloid-related components. **(B)** Boxplots showing specific GO pathways that were upregulated in activated microglia compared to microglia.

To examine the activation status of cellular pathways in MG and aMG cells under different conditions, we performed GSVA on all myeloid-related components, and the results are shown in Figure 8A. We further identified nine GO biological processes that are upregulated in activated microglia under different conditions (Figure 8B): “Positive regulation of myeloid leukocyte differentiation,” “NIK NF-κB signaling,” “CD40 signaling pathway,” “Interleukin 1 beta production,” “Interleukin 18 production,” “Regulation of the insulin receptor signaling pathway,” “Regulation of the extrinsic apoptotic signaling pathway,” “Regulation of the extrinsic apoptotic signaling pathway via death domain receptors,” “Positive regulation of T helper 17 cell differentiation,” “Positive regulation of microphage migration,” “Positive regulation of superoxide dismutase activity,” and “Cellular response to oxygen-containing compound,” suggesting the relevance of these pathways in microglia during the repair process after TBI treatment.

### Validation analysis

The qPCR results corroborated the findings from the single-cell transcriptomics analysis. Consistent with the single-cell RNA-seq data, Ccl2, Tnf, and Grn were significantly upregulated in LPS-treated cells compared to untreated controls. Specifically, Ccl2 expression increased 4.5-fold (*p* = 0.003), reflecting enhanced chemotactic signaling relevant to monocyte recruitment. Tnf expression showed a robust 5.1-fold induction (*p* = 0.001), consistent with classical M1-type pro-inflammatory activation of microglia. Grn expression was upregulated 3.7-fold (*p* = 0.012), supporting its role in modulating microglial differentiation and the neuroimmune environment (Table 1). These results parallel the transcriptional profiles observed in Ly6c^+^ monocytes and MG/aMG subpopulations from the cortex and hippocampus regions in the scRNA-seq data. Collectively, these validation results support the involvement of Ccl2, Tnf, and Grn in early-stage microglial activation and myeloid cell-mediated inflammatory responses relevant to traumatic brain injury (TBI) and potentially aging-associated neuroinflammation.

**Table 1:** Quantitative PCR validation of the selected genes in LPS-stimulated BV2 microglial cells.

Gene	Fold change (LPS vs. control)	*p*-value	Functional role in TBI/neuroinflammation
Ccl2	4.5 ± 0.4	0.003	Monocyte chemoattractant; immune cell recruitment
Tnf	5.1 ± 0.3	0.001	Pro-inflammatory cytokine; M1 polarization marker
Grn	3.7 ± 0.5	0.012	Regulator of microglial differentiation and immune modulation

Values represent mean ± SD from three independent experiments. Fold changes are calculated relative to untreated control cells and normalized to GAPDH expression.

## Discussion

Traumatic brain injury (TBI) induces a cascade of neuroinflammatory events involving resident and peripheral myeloid cells. Using single-cell transcriptomics across blood, cortex, and hippocampal tissues, our study delineated the temporal dynamics of monocyte, macrophage, and microglial populations, revealing both rapid and delayed immune responses. The results not only confirm previous paradigms of post-TBI inflammation but also provide novel insights into LR communication and context-dependent myeloid activation. Importantly, our findings highlight potential intersections between immune signaling, mitochondrial dysfunction, and age-associated vulnerability, offering translational perspectives for therapeutic modulation. We observed a rapid elevation in circulating monocytes (Ly6c^+^.Mon and Treml4^+^.Mon) within 24 h post-TBI, followed by normalization at 7 days. In particular, the enrichment of Treml4^+^.Mon cells supports their role in shaping early inflammatory responses. Treml4 is known to amplify macrophage inflammatory programs (Gonzalez-Cotto et al., 2020), and its transient expansion suggests a controlled pro-inflammatory burst that facilitates tissue repair if timely resolved. This pattern resonates with the broader concept that M1 polarization, while often associated with pathology, may be necessary in the acute phase for the clearance of debris and the initiation of recovery (Kokiko-Cochran and Godbout, 2018). Interestingly, these dynamics also intersect with the biology of aging and mitochondrial function. Aging is associated with a shift toward pro-inflammatory monocyte phenotypes, often referred to as “inflammaging,” which can exacerbate CNS injury outcomes (Youm et al., 2013). Mitochondrial dysfunction in monocytes further drives excessive reactive oxygen species (ROS) production, amplifying inflammatory cascades (Xu et al., 2024). Therefore, the transient Treml4^+^ surge observed here may behave differently in aged or metabolically impaired hosts, a critical consideration for translational relevance.

In both the cortex and hippocampus, macrophages were significantly enriched within 24 h post-TBI, with transcriptomic signatures strongly associated with interferon-γ (IFN-γ) signaling and M1-like polarization (Mühl and Pfeilschifter, 2003). This aligns with prior studies reporting early M1 macrophage predominance after TBI (Mountney et al., 2017). While excessive M1 activity is often detrimental, contributing to secondary injury, its presence during the early phase may facilitate the clearance of cellular debris and the recruitment of reparative immune subsets. The IFN-γ axis is tightly linked to mitochondrial stress responses. Excessive IFN-γ signaling can disrupt mitochondrial membrane potential and promote apoptosis (Jessop et al., 2020). Therefore, the pro-inflammatory macrophage polarization we observed may not only drive cytokine release but also contribute to metabolic reprogramming of CNS cells, linking immune signaling to mitochondrial vulnerability.

Microglial activation emerged as an early hallmark, particularly in the cortex at 24 h post-injury, where upregulated pathways included leukocyte migration, cytokine production (IL1B, IL18), and extrinsic apoptotic signaling. These findings are consistent with classical descriptions of microglial M1-like activation during the early phase of TBI (Madathil et al., 2018). IL1B and IL18 upregulation highlights inflammasome involvement (Ren and Torres, 2009; Hirooka and Nozaki, 2021), which has been tightly linked to mitochondrial dysfunction. Indeed, the activation of NLRP3 inflammasomes is driven by mitochondrial ROS and the release of mitochondrial DNA, providing a mechanistic link between microglial activation and metabolic stress (Zhou et al., 2011). The age context is again relevant. Aging microglia display a primed phenotype with heightened inflammatory responses and impaired resolution, partly due to defective mitochondrial turnover (Streit et al., 2004). Therefore, while young animals may transiently benefit from acute microglial activation, aged individuals could experience prolonged and maladaptive responses.

Through ligand–receptor analysis, we identified three candidate signaling axes mediating cross-talk between activated microglia and circulating monocytes: Ccl2/Ccl7–Ccr2, Tnf–Tnfrsf1b, and Grn–Flna. These pathways highlight mechanisms by which CNS-resident cells orchestrate the recruitment and polarization of peripheral immune subsets. Ccl2 and Ccl7 are canonical chemokines driving myeloid chemotaxis (Volpe et al., 2012; Chen et al., 2017; Zhang et al., 2020). Their upregulation in microglia, coupled with Ccr2 expression in circulating Ly6c^+^.Mon cells, underscores a well-established recruitment axis. Importantly, Ccl2-mediated chemotaxis has been shown to depend on mitochondrial ATP release, suggesting metabolic coupling between immune signaling and bioenergetics (Böttcher et al., 2019). Similarly, the Tnf–Tnfrsf1b interaction suggests a bidirectional polarization loop. TNF-α not only promotes M1 polarization (Wu et al., 2015; Degboé et al., 2019) but also triggers mitochondrial dysfunction via NF-κB-driven metabolic reprogramming (West et al., 2011). In this sense, TNF may serve as a nexus linking inflammatory amplification to mitochondrial injury. Finally, Grn–Flna signaling was identified as a delayed axis, upregulated at day 7 in microglia and monocytes. Granulin plays roles in macrophage differentiation (Ong et al., 2006; Campbell et al., 2021), and its association with Flna suggests involvement in cytoskeletal remodeling. This delayed activation may reflect a transition from acute recruitment to reparative remodeling.

A key conceptual advance from this study is that the transcriptional programs we observed align with broader hallmarks of mitochondrial dysfunction and aging. TBI is known to induce acute mitochondrial damage in neurons and glia, characterized by ROS generation, impaired ATP synthesis, and the release of mitochondrial DNA (Singh et al., 2006). These events, in turn, serve as danger-associated molecular patterns (DAMPs), fueling myeloid activation. Our data, showing upregulation of pro-inflammatory cytokines and chemotactic signals in microglia, fit squarely within this framework. Moreover, the vulnerability of aged individuals to TBI may be explained by impaired mitochondrial resilience and pre-existing inflammaging. In older hosts, monocytes and microglia exhibit diminished capacity for metabolic recovery, leading to chronic neuroinflammation and poor repair outcomes. Therefore, the pathways highlighted here (Treml4, Ccl2/Ccl7, TNF, and Grn) may represent not only acute-response mediators but also amplifiers of long-term maladaptive inflammation in aged or metabolically compromised brains. Our transcriptomics analysis revealed enrichment of oxidative phosphorylation, ROS-associated modules, and mitochondrial stress-linked pathways, consistent with prior reports of post-TBI metabolic vulnerability. These signatures provide indirect but biologically coherent evidence of mitochondrial dysfunction. To maintain the integrity of our current study design, we did not incorporate additional metabolic assays; however, integrating direct mitochondrial functional measurements or validating mitochondrial pathway enrichment using public datasets (e.g., GEO, MSigDB) represents an important next step and aligns with the broader objectives of our ongoing research.

Our study provides novel insights into myeloid dynamics following TBI, yet several areas remain for further exploration. First, while single-cell transcriptomics offers high-resolution mapping of transcriptional programs, mRNA levels do not always perfectly correlate with protein function. Therefore, the absence of functional validation, including cytokine secretion assays, protein quantification, *in vivo* depletion studies, or genetic perturbation, represents an important limitation. Future mechanistic experiments will be required to confirm whether the candidate pathways we identified directly modulate injury responses. Complementary functional studies, such as cytokine secretion assays, *in vivo* depletion models, or protein-level analyses, will help validate and extend these findings. Second, our analysis focused on early time points (24 h and 7 days), capturing acute immune responses. Including intermediate (e.g., 3 days) and longer-term (weeks to months) time points in future studies could provide a more comprehensive view of the immune trajectory and tissue repair. Our experiments were conducted in young adult mice. Since aging is closely linked to mitochondrial dysfunction, immune priming, and altered inflammatory resolution, extending these studies to aged or metabolically compromised animals will be crucial for understanding TBI outcomes across the lifespan. Although the LPS stimulation model is a widely used tool to probe inflammatory responsiveness, it does not fully recapitulate the biomechanical injury, vascular disruption, or metabolic stress associated with TBI. LPS exposure may exaggerate pro-inflammatory polarization and therefore should be interpreted cautiously when extrapolating to *in vivo* injury contexts. Integrating LPS-independent validation models in future research will be essential to strengthen causal interpretation. Our LR analysis identified key candidate pathways, such as Ccl2–Ccr2, Tnf–Tnfrsf1b, and Grn–Flna, that likely orchestrate communication between CNS-resident and peripheral myeloid cells. Future experimental validation, including pathway inhibition or knockout models, will help establish causal roles and potentially reveal therapeutic targets. Collectively, these limitations highlight opportunities for further mechanistic and translational studies while underscoring the strength of our single-cell framework in mapping early myeloid responses after TBI.

## Conclusion

Our study conducted a comprehensive single-cell transcriptomics analysis of myeloid-related immune responses following TBI, revealing dynamic changes in cellular composition, gene expression, and activation states across different tissues. We identified a transient increase in Treml4^+^ monocytes in circulation, early macrophage accumulation in the brain, and activation of microglia enriched in pro-inflammatory pathways, suggesting a coordinated immune response that may contribute to recovery. In addition, our analysis of LR interactions highlights potential mechanisms through which activated microglia regulate peripheral myeloid cell recruitment and polarization. Although further functional validation is needed, these findings enhance our understanding of TBI-induced immune modulation and provide a foundation for future therapeutic strategies aimed at targeting myeloid activation to improve recovery outcomes.

## Data Availability

The original contributions presented in the study are included in the article/supplementary material, further inquiries can be directed to the corresponding authors.

## Footnotes

1
www.ncbi.nlm.nih.gov/geo

2
www.uniprot.org

## References

[ref1] An J. Chen B. Kang X. Zhang R. Guo Y. Zhao J. . (2020). Neuroprotective effects of natural compounds on LPS-induced inflammatory responses in microglia. Am. J. Transl. Res. 12, 2353–2378. 32655777 PMC7344058

[ref2] Arneson D. Zhang G. Ahn I. S. Ying Z. Diamante G. Cely I. . (2022). Systems spatiotemporal dynamics of traumatic brain injury at single-cell resolution reveals humanin as a therapeutic target. Cell. Mol. Life Sci. 79:480. doi: 10.1007/s00018-022-04495-9, 35951114 PMC9372016

[ref3] Blennow K. Brody D. L. Kochanek P. M. Levin H. McKee A. Ribbers G. M. . (2016). Traumatic brain injuries. Nat. Rev. Dis. Primers 2:16084. doi: 10.1038/nrdp.2016.84, 27853132

[ref4] Böttcher C. Schlickeiser S. Sneeboer M. A. M. Kunkel D. Knop A. Paza E. . (2019). Human microglia regional heterogeneity and phenotypes determined by multiplexed single-cell mass cytometry. Nat. Neurosci. 22, 78–90. doi: 10.1038/s41593-018-0290-2, 30559476

[ref5] Campbell C. A. Fursova O. Cheng X. Snella E. McCune A. Li L. . (2021). A zebrafish model of granulin deficiency reveals essential roles in myeloid cell differentiation. Blood Adv. 5, 796–811. doi: 10.1182/bloodadvances.2020003096, 33560393 PMC7876888

[ref6] Chen C. Y. Fuh L. J. Huang C. C. Hsu C. J. Su C. M. Liu S. C. . (2017). Enhancement of CCL2 expression and monocyte migration by CCN1 in osteoblasts through inhibiting miR-518a-5p: implication of rheumatoid arthritis therapy. Sci. Rep. 7:421. doi: 10.1038/s41598-017-00513-0, 28341837 PMC5428676

[ref7] Colonna M. Butovsky O. (2017). Microglia function in the central nervous system during health and neurodegeneration. Annu. Rev. Immunol. 35, 441–468. doi: 10.1146/annurev-immunol-051116-052358, 28226226 PMC8167938

[ref8] D’Mello C. Le T. Swain M. G. (2009). Cerebral microglia recruit monocytes into the brain in response to tumor necrosis factoralpha signaling during peripheral organ inflammation. J. Neurosci. 29, 2089–2102. doi: 10.1523/jneurosci.3567-08.2009, 19228962 PMC6666330

[ref9] Degboé Y. Rauwel B. Baron M. Boyer J. F. Ruyssen-Witrand A. Constantin A. . (2019). Polarization of rheumatoid macrophages by TNF targeting through an IL-10/STAT3 mechanism. Front. Immunol. 10:3. doi: 10.3389/fimmu.2019.00003, 30713533 PMC6345709

[ref10] Fani Maleki A. Rivest S. (2019). Innate immune cells: monocytes, monocyte-derived macrophages and microglia as therapeutic targets for Alzheimer’s disease and multiple sclerosis. Front. Cell. Neurosci. 13:355. doi: 10.3389/fncel.2019.00355, 31427930 PMC6690269

[ref11] Gonzalez-Cotto M. Guo L. Karwan M. Sen S. K. Barb J. Collado C. J. . (2020). TREML4 promotes inflammatory programs in human and murine macrophages and alters atherosclerosis lesion composition in the apolipoprotein E deficient mouse. Front. Immunol. 11:397. doi: 10.3389/fimmu.2020.00397, 32292401 PMC7133789

[ref12] Gschwandtner M. Derler R. Midwood K. S. (2019). More than just attractive: how CCL2 influences myeloid cell behavior beyond chemotaxis. Front. Immunol. 10:2759. doi: 10.3389/fimmu.2019.02759, 31921102 PMC6923224

[ref13] Hänzelmann S. Castelo R. Guinney J. (2013). GSVA: gene set variation analysis for microarray and RNA-seq data. BMC Bioinformatics 14:7. doi: 10.1186/1471-2105-14-7, 23323831 PMC3618321

[ref14] Hao Y. Hao S. Andersen-Nissen E. Mauck W. M. 3rd Zheng S. Butler A. . (2021). Integrated analysis of multimodal single-cell data. Cell 184, 3573–3587. doi: 10.1016/j.cell.2021.04.048 34062119 PMC8238499

[ref15] Hirooka Y. Nozaki Y. (2021). Interleukin-18 in inflammatory kidney disease. Front. Med. 8:639103. doi: 10.3389/fmed.2021.639103, 33732720 PMC7956987

[ref16] Hongxia L. Yuxiao T. Zhilei S. Yan S. Yicui Q. Jiamin S. . (2019). Zinc inhibited LPS-induced inflammatory responses by upregulating A20 expression in microglia BV2 cells. J. Affect. Disord. 249, 136–142. doi: 10.1016/j.jad.2019.02.041, 30772740

[ref17] Jessen K. R. (2004). Glial cells. Int. J. Biochem. Cell Biol. 36, 1861–1867. doi: 10.1016/j.biocel.2004.02.023, 15203098

[ref18] Jessop F. Buntyn R. Schwarz B. Wehrly T. Scott D. Bosio C. M. (2020). Interferon gamma reprograms host mitochondrial metabolism through inhibition of complex II to control intracellular bacterial replication. Infect. Immun. 88:e00744-19. doi: 10.1128/iai.00744-19, 31740527 PMC6977132

[ref19] Kettenmann H. Kirchhoff F. Verkhratsky A. (2013). Microglia: new roles for the synaptic stripper. Neuron 77, 10–18. doi: 10.1016/j.neuron.2012.12.023, 23312512

[ref20] Kim J. Y. Kim N. Yenari M. A. (2015). Mechanisms and potential therapeutic applications of microglial activation after brain injury. CNS Neurosci. Ther. 21, 309–319. doi: 10.1111/cns.12360, 25475659 PMC4376565

[ref21] Kokiko-Cochran O. N. Godbout J. P. (2018). The inflammatory continuum of traumatic brain injury and Alzheimer’s disease. Front. Immunol. 9:672. doi: 10.3389/fimmu.2018.00672, 29686672 PMC5900037

[ref22] Lampron A. Elali A. Rivest S. (2013). Innate immunity in the CNS: redefining the relationship between the CNS and its environment. Neuron 78, 214–232. doi: 10.1016/j.neuron.2013.04.005, 23622060

[ref23] Madathil S. K. Wilfred B. S. Urankar S. E. Yang W. Leung L. Y. Gilsdorf J. S. . (2018). Early microglial activation following closed-head concussive injury is dominated by pro-inflammatory M-1 type. Front. Neurol. 9:964. doi: 10.3389/fneur.2018.00964, 30498469 PMC6249371

[ref24] Mountney A. Boutté A. M. Cartagena C. M. Flerlage W. F. Johnson W. D. Rho C. . (2017). Functional and molecular correlates after single and repeated rat closed-head concussion: indices of vulnerability after brain injury. J. Neurotrauma 34, 2768–2789. doi: 10.1089/neu.2016.4679, 28326890

[ref25] Mühl H. Pfeilschifter J. (2003). Anti-inflammatory properties of pro-inflammatory interferon-gamma. Int. Immunopharmacol. 3, 1247–1255. doi: 10.1016/s1567-5769(03)00131-0, 12890422

[ref26] Ong C. H. He Z. Kriazhev L. Shan X. Palfree R. G. Bateman A. (2006). Regulation of progranulin expression in myeloid cells. Am. J. Physiol. Regul. Integr. Comp. Physiol. 291, R1602–R1612. doi: 10.1152/ajpregu.00616.2005, 16873554

[ref27] Ott L. McClain C. J. Gillespie M. Young B. (1994). Cytokines and metabolic dysfunction after severe head injury. J. Neurotrauma 11, 447–472. doi: 10.1089/neu.1994.11.447, 7861440

[ref28] Ren K. Torres R. (2009). Role of interleukin-1beta during pain and inflammation. Brain Res. Rev. 60, 57–64. doi: 10.1016/j.brainresrev.2008.12.020, 19166877 PMC3076185

[ref29] Schafflick D. Xu C. A. Hartlehnert M. Cole M. Schulte-Mecklenbeck A. Lautwein T. . (2020). Integrated single cell analysis of blood and cerebrospinal fluid leukocytes in multiple sclerosis. Nat. Commun. 11:247. doi: 10.1038/s41467-019-14118-w, 31937773 PMC6959356

[ref30] Shechter R. Raposo C. London A. Sagi I. Schwartz M. (2011). The glial scar-monocyte interplay: a pivotal resolution phase in spinal cord repair. PLoS One 6:e27969. doi: 10.1371/journal.pone.0027969, 22205935 PMC3244386

[ref31] Singh I. N. Sullivan P. G. Deng Y. Mbye L. H. Hall E. D. (2006). Time course of post-traumatic mitochondrial oxidative damage and dysfunction in a mouse model of focal traumatic brain injury: implications for neuroprotective therapy. J. Cereb. Blood Flow Metab. 26, 1407–1418. doi: 10.1038/sj.jcbfm.9600297, 16538231

[ref32] Stark C. Breitkreutz B. J. Reguly T. Boucher L. Breitkreutz A. Tyers M. (2006). BioGRID: a general repository for interaction datasets. Nucleic Acids Res. 34, D535–D539. doi: 10.1093/nar/gkj109, 16381927 PMC1347471

[ref33] Streit W. J. Sammons N. W. Kuhns A. J. Sparks D. L. (2004). Dystrophic microglia in the aging human brain. Glia 45, 208–212. doi: 10.1002/glia.10319, 14730714

[ref34] Tran H. T. N. Ang K. S. Chevrier M. Zhang X. Lee N. Y. S. Goh M. . (2020). A benchmark of batch-effect correction methods for single-cell RNA sequencing data. Genome Biol. 21:12. doi: 10.1186/s13059-019-1850-9, 31948481 PMC6964114

[ref35] Trapnell C. Cacchiarelli D. Grimsby J. Pokharel P. Li S. Morse M. . (2014). The dynamics and regulators of cell fate decisions are revealed by pseudotemporal ordering of single cells. Nat. Biotechnol. 32, 381–386. doi: 10.1038/nbt.2859, 24658644 PMC4122333

[ref36] Volpe S. Cameroni E. Moepps B. Thelen S. Apuzzo T. Thelen M. (2012). CCR2 acts as scavenger for CCL2 during monocyte chemotaxis. PLoS One 7:e37208. doi: 10.1371/journal.pone.0037208, 22615942 PMC3355119

[ref37] West A. P. Brodsky I. E. Rahner C. Woo D. K. Erdjument-Bromage H. Tempst P. . (2011). TLR signalling augments macrophage bactericidal activity through mitochondrial ROS. Nature 472, 476–480. doi: 10.1038/nature09973, 21525932 PMC3460538

[ref38] Wu T. Hu E. Xu S. Chen M. Guo P. Dai Z. . (2021). clusterProfiler 4.0: a universal enrichment tool for interpreting omics data. Innovation 2:100141. doi: 10.1016/j.xinn.2021.100141, 34557778 PMC8454663

[ref39] Wu X. Xu W. Feng X. He Y. Liu X. Gao Y. . (2015). TNF-a mediated inflammatory macrophage polarization contributes to the pathogenesis of steroid-induced osteonecrosis in mice. Int. J. Immunopathol. Pharmacol. 28, 351–361. doi: 10.1177/0394632015593228, 26197804

[ref40] Xu M. Wang W. Cheng J. Qu H. Xu M. Wang L. (2024). Effects of mitochondrial dysfunction on cellular function: role in atherosclerosis. Biomed. Pharmacother. 174:116587. doi: 10.1016/j.biopha.2024.116587, 38636397

[ref41] Xu H. Wang Z. Li J. Wu H. Peng Y. Fan L. . (2017). The polarization states of microglia in TBI: a new paradigm for pharmacological intervention. Neural Plast. 2017:5405104. doi: 10.1155/2017/5405104, 28255460 PMC5309408

[ref42] Yang Q. Q. Zhou J. W. (2019). Neuroinflammation in the central nervous system: symphony of glial cells. Glia 67, 1017–1035. doi: 10.1002/glia.23571, 30548343

[ref43] Youm Y. H. Grant R. W. McCabe L. R. Albarado D. C. Nguyen K. Y. Ravussin A. . (2013). Canonical Nlrp3 inflammasome links systemic low-grade inflammation to functional decline in aging. Cell Metab. 18, 519–532. doi: 10.1016/j.cmet.2013.09.010, 24093676 PMC4017327

[ref44] Zhang M. Yang W. Wang P. Deng Y. Dong Y. T. Liu F. F. . (2020). CCL7 recruits cDC1 to promote antitumor immunity and facilitate checkpoint immunotherapy to non-small cell lung cancer. Nat. Commun. 11:6119. doi: 10.1038/s41467-020-19973-6, 33257678 PMC7704643

[ref45] Zhou R. Yazdi A. S. Menu P. Tschopp J. (2011). A role for mitochondria in NLRP3 inflammasome activation. Nature 469, 221–225. doi: 10.1038/nature09663, 21124315

